# Reflections and New Perspectives on Face Cognition as a Specific Socio-Cognitive Ability

**DOI:** 10.3390/jintelligence9020030

**Published:** 2021-06-11

**Authors:** Kristina Meyer, Werner Sommer, Andrea Hildebrandt

**Affiliations:** 1 Charité—Universitätsmedizin Berlin, Corporate Member of Freie Universität Berlin and Humboldt-Universität zu Berlin, Psychiatric University Hospital Charité at St. Hedwig Hospital, Große Hamburger Str. 5-11, 10115 Berlin, Germany; 2 Institut für Psychologie, Humboldt-Universität zu Berlin and Department of Psychology, Zhejiang Normal University, Jinhua 321004, China; werner.sommer@cms.hu-berlin.de; 3 Department of Psychology, Carl von Ossietzky Universität Oldenburg and the Research Center Neurosensory Science, Carl von Ossietzky Universität Oldenburg, 26129 Oldenburg, Germany; andrea.hildebrandt@uni-oldenburg.de

**Keywords:** socio-cognitive abilities, face cognition, face specificity, speed and accuracy

## Abstract

The study of socio-cognitive abilities emerged from intelligence research, and their specificity remains controversial until today. In recent years, the psychometric structure of face cognition (FC)—a basic facet of socio-cognitive abilities—was extensively studied. In this review, we summarize and discuss the divergent psychometric structures of FC in easy and difficult tasks. While accuracy in difficult tasks was consistently shown to be face-specific, the evidence for easy tasks was inconsistent. The structure of response speed in easy tasks was mostly—but not always—unitary across object categories, including faces. Here, we compare studies to identify characteristics leading to face specificity in easy tasks. The following pattern emerges: in easy tasks, face specificity is found when modeling speed in a *single task*; however, when modeling speed across *multiple, different* easy tasks, only a unitary factor structure is reported. In difficult tasks, however, face specificity occurs in both single task approaches and task batteries. This suggests different cognitive mechanisms behind face specificity in easy and difficult tasks. In easy tasks, face specificity relies on isolated cognitive sub-processes such as face identity recognition. In difficult tasks, face-specific and task-independent cognitive processes are employed. We propose a descriptive model and argue for FC to be integrated into common taxonomies of intelligence.

## 1. Socio-Cognitive Abilities 

The study of socio-cognitive abilities emerged within intelligence research more than a century ago when Thorndike coined the term social intelligence and defined it as the “ability to understand and manage men and women, boys and girls—to act wisely in human relations” (Thorndike 1920, p. 228). Vernon (1933, p. 44) later substantiated this rather broad definition was by suggesting that social intelligence refers to the “ability to get along with people in general, social technique or ease in society, knowledge of social matters, susceptibility to stimuli from other members of a group, as well as insight into the temporary moods or underlying personality traits of strangers”. In other words, social intelligence was thought to encompass a range of specific perceptual and higher-order cognitive abilities that are necessary to successfully navigate through social situations. However, there was no consensus in the field on whether social abilities are specific above and beyond academic skills, including well-established facets of the contemporary cognitive ability taxonomies. For instance, Wechsler believed that “social intelligence is just general intelligence applied to social situations” (Wechsler 1958, p. 75). These different viewpoints exemplify a fundamental debate in cognitive psychology of differential individual differences concerning the specificity of abilities.

Investigating whether cognitive abilities, such as verbal fluency and mathematic reasoning, are dissociated from each other is relevant for establishing the psychometric structure of intelligence. For example, O’Sullivan and Guilford (1975) applied an extensive set of tasks, including verbal and figural reasoning, creativity and social intelligence assessments, and derived a six-factor model of social intelligence. Nevertheless, their proposal was not integrated by Carroll and Cattell-Horn (Carroll 1993; McGrew 2009), who proposed the most comprehensive and today widely accepted (Keith and Reynolds 2010) nomological net of cognitive abilities,^1^ the three-stratum model or Cattell-Horn–Carroll (CHC) model (McGrew 2009; Schneider and McGrew 2012). According to this model, cognitive abilities are grouped hierarchically with a general factor of intelligence (*g*) at the top layer called Stratum III. Below the general factor, there is Stratum II to which domain-specific abilities belong. Stratum I encompasses performance in specific tasks. More recent approaches aiming to conclude with a taxonomy of socio-cognitive abilities, along with their psychometric structure, arose from social and cognitive psychology. In line with the terminology of the field, in a recent review, Happé et al. (2017) used the widely accepted overarching term social cognition and defined it as “the processing of stimuli relevant to understanding agents and their interactions” (p. 244). 

The study of social stimulus processing has received much attention in neurocognitive psychology (Barton 2008; Duchaine and Yovel 2015; Kanwisher 2010; Young et al. 1993). These studies focus mostly on faces, arguing that face processing is a crucial, basic facet of socio-cognitive abilities. However, despite the multitude of experimental, developmental and clinical studies on face processing, its nomological net was only recently addressed from an individual differences perspective (Gauthier et al. 2005; Herzmann et al. 2010; Kaltwasser et al. 2014; McGugin et al. 2016; Meyer et al. 2019a, 2021; Nowparast Rostami et al. 2017). 

### Face Cognition as a Basic Socio-Cognitive Ability

The term Face Cognition (FC) describes a set of specific abilities related to the processing of faces, including face perception and face memory (Chernorizov et al. 2016; Wilhelm et al. 2007, 2010). Faces convey different types of information. These are not readily available from other sources, but highly relevant to human social interaction. According to cognitive and neurocognitive models of face processing (Bruce and Young 1986; Haxby et al. 2000; Schweinberger and Burton 2011), facial information is differentiated into invariant and changeable aspects. Invariant facial information encompasses facial feature configurations from which identity, age or gender can be derived (e.g., Cross et al. 1971). Changeable aspects of faces encompass emotional expressions, gaze direction and facial speech (Bernstein and Yovel 2015; Said et al. 2010), to name a few. In sum, FC refers to abilities necessary to process all these types of facial information, which may turn out to be specific in terms of individual differences. 

The study of individual differences in FC among non-clinical adult populations has received increasing attention during the past decade (Wilmer 2017). Wilmer et al. (2014, p. 1) referred to FC as a “model specific ability” because understanding the cognitive mechanisms^2^ behind face specificity is not only valuable in itself. It can also serve as “informative case study” (Kanwisher and Yovel 2006, p. 2123) to provide important directions for research on the neurocognitive mechanisms of other more or less specific cognitive abilities. The literature refers to FC as a multidimensional facet of social cognition (Herzmann et al. 2008; Wilhelm et al. 2010). The study of FC specificity from an individual differences perspective not only helps understanding the mechanisms underlying domain-specific cognitive abilities, but is also imperative due to the crucial role of facial information in everyday life. 

In this focused review, we aim to summarize and integrate recent discoveries pertaining to the specificity of processing invariant aspects of human faces, such as identity, the spatial organization of facial features, or gender. Further, we discuss how FC can be integrated into the CHC-model of intelligence, which constitutes the most influential nomological net of cognitive abilities today (McGrew 2009). Previous literature suggests that at least some aspects of the ability to process faces as compared to other stimuli is highly specific, but there are also inconsistencies and loose ends. Particularly, performance on different face processing tasks reveals a different psychometric structure in easy relative to difficult tasks. After (1) briefly summarizing evidence on face specificity from different scientific viewpoints, we (2) elaborate the individual differences perspective on FC, and (3) discuss inconsistent findings on its specificity in difficult (accuracy) tasks but not in easy (speed) tasks. Importantly, we aim to (4) understand and explain these inconsistent patterns of results by means of theoretical contemplation and (5) explore the conditions giving rise to FC specificity. Finally, we (6) suggest a neurocognitive model of face specificity considering both accuracy and speed tasks and (7) provide recommendations for future studies. 

These different sections of the review serve the purpose of addressing two overarching questions. Firstly, is there face specificity in performance measures in difficult and easy tasks, and, if so, which rules determine whether face specificity arises? Secondly, once we understand the psychometric structure of FC in easy and difficult tasks, how should we integrate these abilities into the nomological net of cognitive abilities, such as the CHC model (McGrew 2009)? 

## 2. How Special Are Faces? 

In the following, we briefly elucidate how face processing specificity is investigated from the perspectives of different psychological disciplines. We first distinguish between different operationalizations of face specificity and then conclude by carving out an operationalization of specificity from an individual differences perspective, which we then adhere to in the remainder of the article. 

From an experimental perspective, specificity is demonstrated by stimulus-dependent effects of task manipulations. Generally speaking, if a certain manipulation of a stimulus belonging to one category affects task performance differently as compared to stimuli from another category, specificity is inferred. In the FC literature, a multitude of face-specific experimental effects have been described. Well-known paradigms such as the inversion task (Farah et al. 1995; Valentine 1988), the composite task (Young et al. 1987) or the part-whole task (Tanaka and Farah 1993) tap into configural processing and reveal stronger effects when applied to facial stimuli as compared to other visual objects, such as houses, vehicles, animals or household objects. These observations have fostered theories of holistic processing of faces, which stand in contrast to the more feature-based processing of other visual objects (Richler and Gauthier 2014; Tanaka and Simonyi 2016). 

In neurocognitive psychology, specificity refers to the existence of specialized neural structures preferentially processing a limited set of stimuli or to the elicitation of a consistent pattern of brain activation upon presenting participants with a given stimulus type. Neurocognitive specificity of social stimulus processing is supported by findings indicating selective impairment of social processes such as face recognition (Bodamer 1947; Karmiloff-Smith et al. 1995) or voice recognition (Garrido et al. 2009; Neuner and Schweinberger 2000), following brain damage (Shallice 1988) or in developmental disorders (Karmiloff-Smith 1992). More recent research on neurocognitive specificity emphasizes specific spatial and temporal patterns of brain activation in response to social stimuli, such as faces (Bentin et al. 1996; Eimer 2000; Kanwisher 2010; Kanwisher et al. 1997; Kaufmann et al. 2009; Neumann and Schweinberger 2008), voices and other social stimuli (for a review see Schweinberger and Schneider 2014). Both phenomena, functional activation in specific brain areas and selective face processing impairments, are evidence for face specificity from a neurocognitive point of view (Barton 2008; Duchaine and Yovel 2015; Kanwisher 2010; Young et al. 1993). 

In behavior genetics, molecular and developmental psychology, human abilities and traits are specific if they are heritable independently of other characteristics, if specific genetic effects exist and if they demonstrate a unique developmental trajectory. In twin studies, heritability of FC ranges between 76% and 97% in an older sample and 68% to 87% in a younger sample (Wilmer et al. 2010; Shakeshaft and Plomin 2015). There is also some evidence for genetic markers of FC abilities (Hildebrandt et al. 2016; Kiy et al. 2013; Quinones Sanchez et al. 2021; Skuse et al. 2014; Verhallen et al. 2017). Moreover, the psychometric structure of FC is subject to developmental changes across the life span that are not fully explainable by developmental changes in general cognitive abilities, perceptual acuity or physical health (Hildebrandt et al. 2011, 2013; Olderbak et al. 2015; Turano and Viggiano 2017).

Considering human abilities from the perspective of evolutionary psychology, researchers argue that evolution shaped our cognitive functions. Thus, observing which cognitive functions are specific allows evolutionary psychologists to draw conclusions on the specific demands imposed on our ancestors (Cosmides and Tooby 1994). In case of FC, a wide array of specific behavioral and biological mechanisms has evolved (Duchaine and Yovel 2015; Kanwisher and Yovel 2006; Wilmer et al. 2014). This remarkable specificity capitalizes on the functional relevance of FC (Adolphs 2009; Dunbar 1998, 2003; Dunbar and Shultz 2007), suggesting a crucial role of social communication in the evolution of humans. This even extends to the idea that general intelligence may be a (partial) by-product of socio-cognitive abilities (Dunbar 2003).

**Figure 1:**
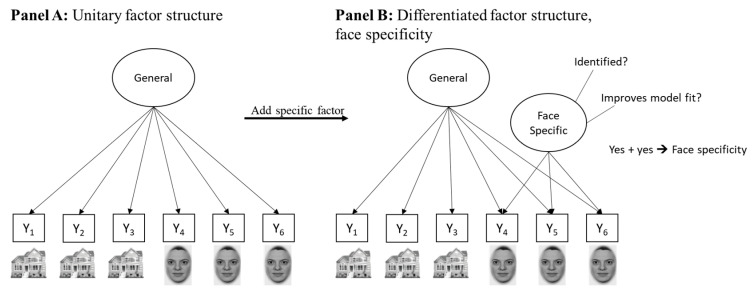
Schematic illustration of a model depicting a unitary factor structure (**Panel A**) and a factor structure differentiated for stimulus content (**Panel B**). The model in Panel B suggests face-specific abilities underlying the performance in tasks Y_1_–Y_6_. Y_1_–Y_6_—performance in six tasks or task trials with houses (Y_1_–Y_3_) or faces (Y_4_–Y_6_) as stimuli.

In individual differences research, stable person characteristics influencing the person’s behavior in a task are investigated. Specificity from the perspective of individual differences describes the occurrence of a distinctive pattern of between-person correlations in task performance. If task responses across multiple individuals, for example the answers given in a questionnaire or the performance in a cognitive task, share a considerable amount of variance with their behavioral responses in other tasks, a common underlying cause is assumed. Performances in face tasks share more common variance with each other than with non-face tasks. Structural equation modeling (SEM) is a suitable and established method for investigating such patterns of individual differences. In SEM, if behavioral performance scores across different tasks or task trials covary, a common latent factor captures the covariation. The latent factor represents the test-taking person’s characteristics that influence performance in all tasks or trials, for example general cognitive ability. In Figure 1, the general latent factor captures this common variance across task performance indicators Y_1_–Y_6_. The indicators Y_1_–Y_6_ might represent six different tasks or, alternatively, six different trial parcels from the same task. If a subset of these tasks, like Y_5_–Y_6_ in Figure 1, shares additional variance that is not fully accounted for by the general factor, meaning that they are still correlated after the variance explained by the general factor is accounted for, it suggests that another underlying specific trait influences performance in Y_5_–Y_6_. This is tested in SEM by adding a specific factor to a model. It is then investigated whether the specific factor is identified (i.e., whether its variance is different from zero) and whether adding the factor significantly improves the model fit. If both conditions are met, the model suggests that a face-specific cognitive ability influences task performance in addition to a general cognitive ability. 

We emphasize that some of the evidence summarized above is not considered unique to face processing according to the expertise hypothesis (Gauthier 2018; Tanaka and Gauthier 1997). The expertise hypothesis suggests that faces are not special due to an innate, evolutionary importance of faces as social cues. Instead, this theory suggests that extensive and repeated exposure to a stimulus class and practice in performing cognitive operations on these stimuli leads to specificity, and that this holds true for many kinds of stimuli, e.g., cars or birds for car and bird experts, respectively. From this point of view, faces are not special by evolution, but happen to represent a stimulus type that most adults grow up to become experts in (Bukach et al. 2006; Tanaka and Gauthier 1997). Proponents of the expertise hypothesis would apply similar specificity operationalizations as mentioned above, investigating neuropsychological data (Burns et al. 2019), experimental data (Gauthier and Bukach 2007) or individual differences in processing objects of expertise (Gauthier et al. 2014). Importantly, the present review is not intended to favor or contradict the expertise hypothesis, as it is not preoccupied with unraveling the causes underlying face specificity. In other words, we aim to shed light on *how* face processing is specific, not *why* it is specific. The expertise hypothesis, however, focuses on the *why* of face specificity. 

In summary, multiple psychological disciplines demonstrate specialness of processing faces, which seems to differ from the processing of other stimuli. These observations indicate that face-specific mechanisms are at work, be these neural, cognitive or genetic mechanisms, influencing humans’ responses to faces in a specific way relative to other stimuli. This review is dedicated to unraveling which mechanisms give rise to face specificity from an individual differences perspective. In the following, we summarize individual differences evidence in favor of face specificity. Throughout the article, we refer to face-specific task performance or face-specific cognitive abilities as outlined in Figure 1 when using the term face specificity. 

### Individual Differences in Face Cognition 

Between-person variability is modeled to investigate patterns of covariances and make inferences about convergent and divergent cognitive abilities. In this line of research, commonly applied statistical approaches are exploratory and confirmatory factor analyses and SEMs. With SEM, one can infer the structure of cognitive abilities by investigating systematic patterns of covariation between different measures. Utilizing these statistical methods has allowed researchers to map the nomological net of intelligence (e.g., Carroll 1993). Most contemporary theories of intelligence assume domain-specific and general cognitive abilities operating in an interrelated manner, a psychometric structure that is best represented by hierarchical or higher-order models with a general factor at the top (Carroll 1993; Flanagan and Dixon 2014; Flanagan and McGrew 1998; Schneider and McGrew 2012; Schulze 2005).

In the growing body of studies investigating the covariance structure of FC (Gauthier 2018; Turano and Viggiano 2017; Wilmer 2017; Yovel et al. 2014; Wilhelm et al. 2010), the variance shared between FC and general cognitive ability ranges from as little as 3% (Wilmer et al. 2010) up to 31% (Wilhelm et al. 2010). Moreover, FC has a multidimensional structure. On a higher hierarchy level, general cognitive abilities are located. The lower levels of the hierarchy encompass specific face-related abilities such as perception and memory of facial identity (Duchaine and Nakayama 2006; Farah et al. 1998; Kanwisher 2000; Wilhelm et al. 2010; Young et al. 1987) and facially expressed emotions (Calder et al. 2000; Calder and Young 2005; Hildebrandt et al. 2015a, 2015b; Neta and Whalen 2011; Olderbak et al. 2019). 

Aiming to discover the psychometric structure of cognitive abilities, researchers construct tasks along different dimensions of task characteristics. According to facet theory (Guttman 1954; Süß and Beauducel 2005; Oberauer et al. 2000, 2003), such task characteristics comprise stimulus *content* and task *operation*. Content describes the types of stimuli, for example faces relative to houses, animals or other non-face objects. Operation refers to the task requirements, for example memorizing and recalling or recognizing stimuli in a memory task or differentiating between stimuli in a perception task. Facet theory holds that the more characteristics are shared by two tasks, the more common inter-individual variance will be observed in the resulting performance measures. Moreover, task difficulty is an important characteristic as well. A number of studies have attempted to disentangle these dimensions and understand the factors that may influence the dimensionality of FC ability. Many of these efforts draw their inspiration from the study of Wilhelm et al. (2010). Wilhelm et al. (2010) varied task operations (memory vs. perception) and task difficulty (speed vs. accuracy), and investigated individual differences in accuracy and speed measures of FC. Gradually, a coherent picture of the FC ability structure emerges, which we outline in the present review. We focus on the distinction between accuracy and speed measures of FC performance because there are some puzzling results concerning this distinction. Our aim is to analyze conflicting findings and conclude on the psychometric structure of speed and accuracy measures of FC.

## 3. On Accuracy- and Speed-Related Face Cognition Abilities 

Carroll (1993, p. 644) stated: “If any broad taxonomic classification of cognitive ability factors were to be formulated, […] it might be one based on the distinction between level and speed.” The study of cognitive abilities typically encompasses performance measures from difficult and easy tasks. In difficult tasks, most individuals will not be able to solve the tasks completely, irrespective of the time accredited. Performance in these tasks varies in terms of accuracy. In easy tasks, performance is often at ceiling. Thus, response times (RTs) or inverted RTs are usually studied in easy tasks. Taxonomies of cognitive abilities primarily comprise accuracy facets (Carroll 1993; Flanagan and McGrew 1998; Schneider and McGrew 2012), whereas understanding the psychometric structure of processing speed has proven to be more challenging (Danthiir et al. 2005). 

Heeding this dichotomy of easy speed tasks and difficult accuracy tasks, Wilhelm et al. (2010) varied task difficulty and task operation (perception and memory) in two samples (*N* = 151, *N* = 209). They used a total of 11 tasks previously established by Herzmann et al. (2008), see Table 1. In SEMs, operation-specific latent factors of face memory and face perception accounted for accuracy in difficult tasks. Furthermore, FC as measured by accuracy tasks was distinguished from general cognitive abilities, confirming FC as a specific ability. In contrast, such a differentiated factor structure did not emerge for the speed of face and object processing. A single, general latent factor exhaustively explained individual differences in RTs, irrespective of cognitive operations (perception or memory).

### 3.1. Theoretical Accounts of Face Specificity Versus Generality in Accuracy and Speed 

Further studies addressed this surprising divergence between accuracy and speed measures of FC. Before summarizing and discussing their results, it is helpful to reflect on the differentiated structure of FC accuracy and the unitary structure of FC speed from a theoretical point of view. To better understand the mechanisms giving rise to face specificity, we take two theoretical perspectives. Both theoretical perspectives do not originate from the FC literature and could be applied to processing of any other stimuli.

The first theoretical perspective pertains to a differentiated view on cognitive sub-processes. In this framework, it is possible to differentiate between cognitive sub-processes of perception, central cognitive processing (e.g., memory retrieval, decision making) and motor responses (Donders [1868] 1969; Sternberg 1969). Each of these sub-processes comes with a certain time demand and probability of success. Easy tasks likely require relatively little central cognitive processing. Thus, variance in easy task performance (RTs and accuracy) is likely dominated by perceptual and motor response-related processes. In contrast, for difficult tasks, central cognitive processes may drive variance of performance to a higher degree. If this reasoning is correct, the unitary factor structure of speed in easy tasks indicates that perceptual and motor-response-related sub-processes of cognition, which dominate the variance in easy tasks, bear no or minimal face specificity.

**Figure 2:**
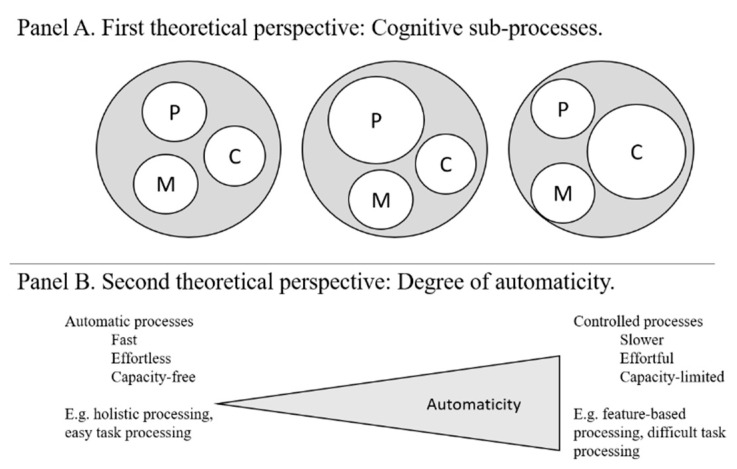
(**Panel A**): Illustration of variance components of cognitive sub-processes needed in different magnitudes depending on the task characteristics. This is to say that tasks rely to different degrees on cognitive sub-processes. Performance variance in a task (grey circle) can be decomposed into variable components related to sub-processes of cognition: P—perceptual processes; C—central cognitive processes; M—motor response-related cognitive processes; remaining grey area representing measurement error and other cognitive processes. (**Panel B**): Illustration of automaticity as a task characteristic.

Consequently, the differentiated factor structure in accuracy tasks is possibly brought forth by the additional central cognitive processes imposed in difficult tasks. More specifically, difficult tasks introduce a larger number of distinct cognitive operations and more sources of potential error than easy tasks. According to this view, face specificity in the accuracy of difficult tasks is a property of central cognitive processes. Figure 2, Panel A illustrates how perceptual, central or motor-related cognitive processes (as indicated by the white circles of varying diameter) dominate the total performance variance (as indicated by the outer circles, including grey areas representing measurement error and other cognitive processes). The relative performance variance depends on the corresponding task demand. For example, central cognitive processes should dominate performance variance in a difficult working memory (right), whereas perceptual processes should dominate a task with aggravated perceptual conditions (middle).

The second perspective relies upon psychological theories proposing that any cognitive process varies along the continuous property of automaticity (LaBerge and Samuels 1974; Evans and Stanovich 2013; Stanovich et al. 2011; see Figure 2, Panel B). Automatic as opposed to controlled cognitive processes are fast, occur in parallel and require little cognitive effort and little or no attention or deliberate control by the individual (Bargh 1982; Hammar 2012). In contrast, controlled processes are capacity-limited, effortful and slow. Individuals can control them deliberately and apply them to new situations. Faces are processed more holistically than other objects, which is thought to indicate more automatic processing (Ćepulić et al. 2020; Cheung and Gauthier 2010). Furthermore, increasing task difficulty diminishes the degree of automaticity in cognitive processing (Becker et al. 2016; Goldhammer et al. 2014). Because automaticity depends on stimulus content and task difficulty, in manipulating both stimulus content and task difficulty, Wilhelm et al. (2010) as well as subsequent studies implicitly also varied the degree of automaticity. Consequently, the previously observed patterns of unitary psychometric structure of speed and differentiated accuracy structure might indicate that face specificity is a byproduct of controlled rather than automatic processing. 

## 4. Conditions of Face Specificity

Now we turn to review empirical studies conducted since the discovery of discrepant factor structures of accuracy and speed measures of FC by Wilhelm et al. (2010). As explained above, Wilhelm et al. (2010) demonstrated that accuracy of FC in easy tasks was differentiated for operations, that is, face memory and face perception were separable abilities. However, in easy tasks, speed of FC was a single unitary factor accounting for RTs in both face memory and face perception performance. The differentiated factor structure for accuracy of FC has been replicated in different data sets (e.g., Hildebrandt et al. 2011; Kiy et al. 2013; Nowparast Rostami et al. 2017; Rotshtein et al. 2007; Wilhelm et al. 2014; Yovel et al. 2014). Furthermore, the factor structure of accuracy tasks is stable across the adult life span (Hildebrandt et al. 2010, 2011, 2013) and sexes (Sommer et al. 2013).

Whereas the differentiated structure of FC accuracy in difficult tasks seems to be a clear and robust finding across studies, waters are muddier in the case of FC speed. As we outline in more detail in the following sections, some studies revealed evidence for a unitary speed factor across faces and non-face objects (Hildebrandt et al. 2010, 2013; Sommer et al. 2013). However, in contrast with these studies, some researchers find face-specific factors of speed in easy tasks (Ćepulić et al. 2018; Hildebrandt et al. 2012; Liu et al. 2017). To carve out which task properties result in face specificity and which do not, in the following, we group the studies according to their conclusions: We start by describing studies reporting a unitary speed factor and continue by introducing those that found a differential speed-related ability structure of FC.

Many of the studies reported below (Table 2) used variants of the tasks first published by Herzmann et al. (2008). Herzmann et al. (2008) provided a battery of easy and difficult perception and memory tasks. The distinction of speed and accuracy tasks followed the psychometric tradition (Carroll 1978) where easy speed tasks and difficult accuracy tasks are distinguished. According to this tradition, the kind of manipulation that makes a task easy or difficult depends on the task. In memory tasks, participants memorize stimuli or parts thereof and are later asked to recognize them. Easy memory tasks require individuals to memorize a single stimulus or a small set of stimuli at a time (as in the *delayed non-matching to sample task*). Difficult memory tasks require memory of a larger number of stimuli (e.g., 30, as in the *acquisition curve* task) or induce a longer delay between encoding and recognition (as in the *decay rate* task). In perception tasks, judgments about similarities across stimuli are common. In easy perception tasks, these are usually obvious after brief observation (as in the *simultaneous matching from different viewpoints* task). Difficult perception tasks either employ small, difficult-to-detect differences (as in the task *facial resemblance*, difficult condition) or require responses under atypical viewing conditions which disrupt holistic processing, for example by inverting the stimuli (as in the task *simultaneous matching of spatially manipulated faces*) by presenting only stimulus parts. In the end, what characterizes a task as easy or difficult is the accuracy rate, which should be 90% or higher for easy tasks and around 75% or lower for difficult tasks (Carroll 1993; Sperling and Dosher 1986).

### 4.1. No Specificity in Speed: Studies Reporting a Unitary Ability Structure in Easy Tasks

Replicating the factor structure reported by Wilhelm et al. (2010), several studies find no specificity for faces in easy speed tasks. Using a test battery consisting of 10 different easy speed tasks, Hildebrandt et al. (2012) found that speed of recognizing facial identity overlapped perfectly with the speed of facial emotion expression recognition in a sample of *N* = 151 young adults. In a study with a large sample (*N* = 448) of participants ranging between 18 and 88 years of age Hildebrandt et al. (2013) revealed that the speed of FC—measured with five different tasks—was perfectly correlated with the speed of object perception. Nowparast Rostami et al. (2017) measured the speed of face and house perception and memory with eight different tasks in *N* = 198 young adults. Replicating previous findings, a single speed factor was enough to explain individual differences in speed performance across all tasks. 

### 4.2. Specificity in Speed: Studies Reporting a Differentiated Ability Structure in Easy Tasks

In contrast to the evidence above, suggesting that a unitary factor accounts for the entire variance in easy face and object tasks, some studies do indeed report face specificity in easy tasks. Ćepulić et al. (2018) asked *N* = 186 healthy young adults to perform a single block-wise learning and recognition task with 10 different object categories, including faces. Each task was administered in a difficult and an easy condition (learning and recognizing 15 vs. 4 stimuli). For the easy conditions, the best fitting model for RTs consisted of a general object recognition factor with specific factors for faces and vehicles. This suggests that speed of face recognition and speed of vehicle recognition are domain-specific abilities beyond general object recognition speed. In another dataset investigating a single face or house recognition task with a difficult and an easy condition (Ouyang et al. 2020), face specificity in RTs of the easy condition also occurred. The repeated findings of face specificity in easy and difficult recognition tasks (Ćepulić et al. 2018; Ouyang et al. 2020) point at a similar factor structure for easy and difficult task performance.

In Meyer et al. (2019a), the EZ diffusion model (Ratcliff 1978; Wagenmakers et al. 2007) was applied to decompose RTs and accuracy scores of *N* = 216 individuals across five easy face and object cognition tasks. The EZ diffusion model allows the derivation of psychologically meaningful parameters, using information from RTs and accuracies simultaneously. Therefore, it provides more sensitive performance measures than RTs and accuracies alone. The EZ diffusion model assumes that an individual, when facing a decision in a two-choice task, accumulates evidence until a certainty threshold is reached. Then, a response is selected. If participants choose to respond more cautiously because, for example, they are instructed to favor accuracy over speed (Voss et al. 2004), these certainty thresholds increase. Such an increase in response caution is reflected in the corresponding threshold parameter of the EZ diffusion model. If there is no face processing-related specificity in easy task performance, there should also be no face specificity in any of the diffusion model parameters. Interestingly, Meyer et al. (2019a) found face specificity in the response caution parameter derived from diffusion modeling, indicating that participants’ tendencies to set the speed-accuracy balance were different for faces and houses. 

Upon detecting face specificity in data from speed tasks, Meyer et al. (2021) explored two possible explanations. As a first explanation, the authors assumed that the cognitive operations (perception vs. memory) might account for the discrepant findings. In studies reporting face specificity in easy tasks (Ćepulić et al. 2018; Meyer et al. 2021; Ouyang et al. 2020), only data from recognition memory tasks had been analyzed. In contrast, studies reporting a unitary speed factor accounting exhaustively for individual differences in speed (Nowparast Rostami et al. 2017; Wilhelm et al. 2010) had used both memory and perception tasks. Therefore, one explanation might be that face specificity occurs in easy recognition tasks, but not in easy perception tasks. 

The second explanation pertained to the difference between single task paradigms vs. task batteries. Studies reporting face specificity in easy tasks had analyzed individual differences in *single task* performance rather than multiple tasks tapping into the same process. Thus, only speed in a single task had been analyzed. This means that a latent factor was estimated to account for the shared variance across multiple trials or blocks of the same task. In studies reporting a unitary speed factor, data from batteries of *multiple tasks* had been jointly modeled. Thus, in these studies, a latent speed factor was estimated based on performance variance across individuals in different speed tasks. Therefore, one might alternatively assume that face specificity hinges upon using single vs. multiple tasks. 

To empirically evaluate these two alternative explanations, Meyer et al. (2021, p. 126) reanalyzed data from Nowparast Rostami et al. (2017). This dataset comprised RTs from eight perception and memory tasks with face and house stimuli in both difficult and easy conditions. Differently from the approach chosen by Nowparast Rostami et al. (2017), Meyer et al. (2021) used SEMs to model individual differences in face and house processing speed separately for each of the eight available tasks. For these single-task models, trials were parceled into three trial sets per task. Average RTs in these item parcels served as indicators for the latent performance speed factors. Previously, the same tasks were jointly modeled, performance in the individual tasks serving as indicators in a common measurement model. If the first explanation held true, Meyer et al. (2021) expected to find face specificity in memory tasks, but not in perception tasks. If the second explanation held true, performance in single tasks should reveal face specificity, irrespective of the cognitive operation. When modeled separately, all memory and perception speed tasks revealed clear face specificity, even though the same data did not reveal face specificity when the multiple task data were modeled jointly (Nowparast Rostami et al. 2017). 

### 4.3. What Differentiates the Studies?

As an interim summary, whereas some studies did not find face specificity in speed task performance, this was the case in two independent data sets (Ćepulić et al. 2018; Meyer et al. 2021). What marks these later studies and differentiates them from the former work reporting a unitary speed factor is their approach of selecting indicators for modeling. Whereas no content specificity could be detected in speed data across multiple tasks, single memory (Ćepulić et al. 2018; Meyer et al. 2021) as well as perception tasks (Meyer et al. 2021) revealed face specificity in performance speed. 

We thus face two different approaches of selecting indicators for SEM. When jointly modeling multiple tasks, we estimate a task-independent psychological construct. The estimated latent variable is generalizable across multiple measurement options of the construct in question. In contrast, when modeling a single task and defining trial blocks of the same task as indicators, we estimate a task-specific latent variable accounting for shared variance across trials or trial blocks. In summary, easy tasks appear to reveal face-specific cognitive processes that lead to a face-specific ability structure. However, face-specific processes remain hidden when performance across multiple tasks requiring different cognitive operations are jointly modeled. 

## 5. Proposal for an Explanatory Model of Face Cognition Specificity 

We now reconcile the psychological theories outlined above with the accumulated evidence for common, face-related specificity in difficult tasks and isolated, task-related face specificity in easy tasks. We argue that neuroscientific findings on FC can help explain the observed distinction. Further, we propose a model to explain existing patterns of results. 

Performance measures that are similar in terms of the cognitive operations and the stimulus categories used share a higher proportion of variance than measures that differ on any or all of these dimensions (Oberauer et al. 2005). This arises for two reasons. First, cognitive processes elicited by a given task (e.g., a memory task) should overlap across different stimulus sets (e.g., faces and houses). Second, cognitive processes elicited by the same stimulus set (e.g., faces) should overlap across tasks (e.g., memory and perception tasks) as well. This pattern becomes visible, for example in studies reporting face-specific memory- and perception-related abilities outlined above, where face memory and face perception are more closely related with each other than with general cognitive abilities (e.g., Wilhelm et al. 2010). Importantly, task difficulty introduces a further dimension of complexity by influencing the degree of automaticity and the quantity and quality of cognitive operations required by the task, as is explicated in the following.

**Figure 3:**
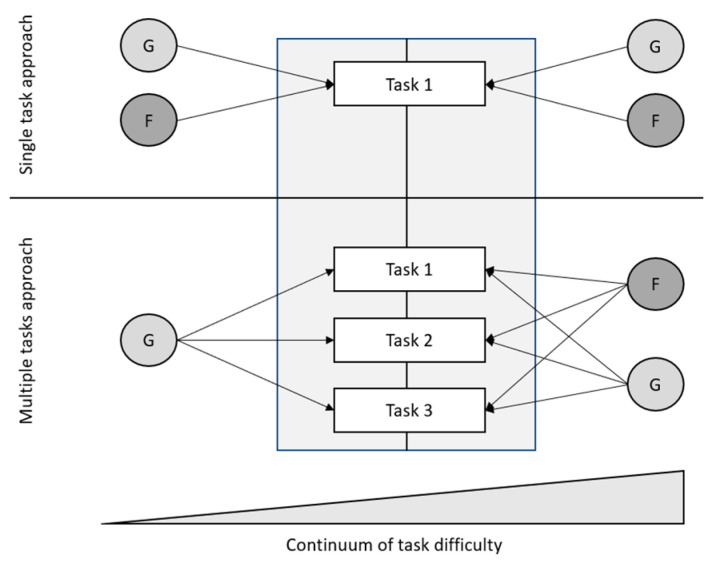
Face specificity in single vs. multiple FC tasks of increasing difficulty. Circular shapes represent underlying abilities (constructs) while angular shapes represent observed performance scores. G—general factor; F—face-specific factor. Task 1–3—these represent possible tasks as proposed in FC research, such as tasks constructed by Herzmann et al. (2008) and applied by Wilhelm et al. (2010). These tasks are not all on the same difficulty level; rather, the tasks position on the easy–hard continuum influences the ratio of task-specific vs. task-independent face processing ability it taps into, represented as face-specific factors in the figure.

All specific easy tasks bear face specificity, which cancels out when tasks are jointly modeled. Possibly, this happens because each task has its own face-specific challenges. In other words, easy tasks activate face-related cognitive operations specific to the task. For example, an easy face perception task with morphed stimuli requires a different face-specific operation than an easy facial resemblance task, although the latter is also classified as a perception task (see Figure 1). The low overlap between these task-specific and face-specific operations results in face specificity in single easy tasks, meaning that multiple trials or blocks of the same tasks become indicators to estimate latent factors. In contrast, when jointly modeling several easy tasks, no face-specific factor emerges that is separable for cognitive speed in general. This is because the face-specific operations across individual tasks do not overlap, and thus do not share variance relative to the common task- and stimulus-independent variance in easy tasks. Importantly, note that this low variance overlap across tasks only occurs for the *face-specific variance component*, not in general. This is to say that correlations between speed tasks in general are very high. However, the face-specific proportions of variance across tasks seem to have little overlap. This explains why studies find face-specific factors of speed in single tasks, but not when jointly modeling multiple easy tasks (see Figure 3).

In difficult tasks, specificity also occurs when jointly modeling multiple tasks, potentially because difficult tasks trigger higher-level face-related abilities, such as face perception and face memory. These higher-level operations overlap more strongly across different types of tasks. Thus, task- and stimulus-specific cognitive operations in easy tasks lead to within-task face specificity in easy and more automatic tasks. Difficult tasks need more deliberate processing and employ across-task face cognition abilities. Thus, with increasing difficulty, the degree of automaticity decreases and the degree of central process employment increases. In other words, face specificity occurs in easy as well as in difficult tasks. The difference is that in easy tasks, face specificity is also task-specific, whereas difficult tasks tap into more task-independent face-specific cognitive abilities. 

Figure 3 is a schematic illustration of the patterns of task- and stimulus-specific operations captured as latent variables underlying performance in single easy FC tasks and task-general face-specific operations underlying performance in difficult FC tasks. In Figure 3, task difficulty represented at the bottom emphasizes that this task property is continuous rather than binary. When face tasks are modified along this continuum by making them easier or more difficult, our model predicts an increase or decrease of correlations between face-specific variance proportions in performance measures across the different tasks. The top half of Figure 3 shows that in given single tasks, performance variance can be decomposed into general and face-specific factors in both easy and difficult tasks. In multiple task approaches, where performance from multiple tasks is jointly modeled (bottom half), performance speed is accounted for by only a general factor, while in difficult tasks, accuracy is explained by a general and a face-specific factor.

Finally, reconciling the explanatory model with the automaticity framework and the cognitive sub-processes framework described earlier (see Section 3.1 and Figure 1), we can conceive of different constellations of automaticity, cognitive sub-processes, and FC. Cognitive sub-processes entail perceptual, central cognitive and motor response-related ones. In any of these sub-processes, task difficulty and individual differences in ability or expertise determine task performance and the degree of automaticity (Horn and Hofer 1992; Roberts and Stankov 1999). This is the case in FC ability, but it might also be for other specific abilities. For example, chess players display highly automatized perceptual and central cognitive operations and are therefore able to process large amounts of chess-related information at once (Reingold et al. 2001). Growing automaticity in motor-related processes may allow individuals to become skilled with complex sports or with musical instruments (Doyon and Benali 2005).

Aside from this, face specificity may be present in any of the three categories of sub-processes. At the perceptual level, face specificity receives support from experimental studies demonstrating holistic face processing (Tanaka and Gordon 2011) and individual differences studies showing specific face perception factors separable from face memory (Wilhelm et al. 2010). Specificity in central cognitive processes affects performance in difficult cognitive tasks of face-specific memory and decision making (e.g., Ćepulić et al. 2018; Meyer et al. 2021). Additionally, the low degree of shared variance between face memory ability and general cognitive ability suggests face-specific central cognitive processes (Wilmer et al. 2010, 2012). Finally, face specificity in motor-related processes might contribute to individual differences in people’s facial emotion processing abilities (Gunnery et al. 2013; Hildebrandt et al. 2015a, 2015b). 

## 6. Neurocognitive Mechanisms Underlying Specificity in Accuracy and Speed

We propose a neurocognitive explanation of the dichotomy in FC specificity patterns summarized above. In easy tasks, the engagement of specialized single neural structures suffices. For example, in order to recognize a well-known familiar face, the occipital face area (OFA) plays a crucial role (Rossion et al. 2003). In this case, no or little higher cognitive evaluation of the stimulus is needed. Thus, for a given easy task, only highly specialized brain areas might be involved, but these might differ from task to task. With increasing task difficulty, more distributed face-related brain areas need to be recruited. Arguably, difficult tasks require multiple cognitive processes, resulting in coordinated activation of multiple brain areas. If these areas are part of the core and extended FC brain networks (e.g., Liu et al. 2020; Meyer et al. 2019b; Wang et al. 2020), this might explain face-specific and task-independent cognitive processing. Importantly, task difficulty is a continuous task characteristic ranging from very easy to very difficult. Hence, we do not suggest that either one or the other neural system is active at a given time point, while the other is not. Instead, brain activity always consists of multiple interrelated processes that occur simultaneously in task-dependent patterns that may gradually shift from easy to difficult task conditions.

In sum, we propose a distinction between easy tasks, processed mainly in individual brain areas such as the fusiform face area (FFA) and the OFA, and difficult tasks, processed by the involvement of a more widespread network. Although there is little evidence to date directly supporting this proposition (e.g., but see Dowdle et al. 2021; Karimi-Rouzbahani et al. 2021), it is compatible with the fMRI literature on neural efficiency. In this literature, functional brain activation of individuals with high and low cognitive abilities has been measured while participants worked on cognitive tasks (Neubauer and Fink 2009). If high-ability individuals solve tasks that are easy for them, a lower amount of cognitive resources was invested. Furthermore, the activated brain areas were usually more posterior and closer to perceptual brain areas (Dunst et al. 2014) as compared to low ability individuals, for whom the same task was difficult. This is similar for language-related areas in easy lexical and speech-related tasks (Zhang and Wang 2007). If an individual is confronted with a more difficult task, this requires the involvement of brain networks related with working memory and higher cognitive processing (Motes et al. 2008; Rypma et al. 2006). The factorial differentiation into accuracy- and speed-related abilities could therefore be a result of efficient domain-specific neural resource allocation. The more difficult the task, the more likely it taps into face-specific, task-independent cognitive abilities, such as face memory or face perception. Finally, an increasing involvement of such task-independent FC abilities and face-specific brain networks also increases the chance to detect specific ability factors across tasks and individuals. 

## 7. Future Directions

Our arguments can be extended to the expertise hypothesis of FC (Diamond and Carey 1986; Tanaka and Gauthier 1997). According to this influential theoretical perspective, specificity is a result of acquired expertise rather than of evolution. Whereas the proponents of the expertise hypothesis argue that most of us are face experts due to our extensive experience with faces, others argue that the heritability (Wilmer et al. 2010; Zhu et al. 2010), the strength of neural selectivity of certain brain areas (Kanwisher 2010; Kanwisher and Yovel 2006), the psychometric structure of FC (Ćepulić et al. 2018) and the quantitative extent of processing specificity for faces (Wilmer et al. 2010, 2012) may not be explainable by expertise alone. As the CONSPEC and CONLERN hypothesis implies (Morton and Johnson 1991), FC is a specific ability by both nature as well as nurture—see also a recent study by Quinones Sanchez et al. (2021), demonstrating that nature and nurture shape structural connectivity in the face processing brain network. Thus, both biological and environmental factors determine the degree of specificity. A possible mechanism of the environmental influence on face specificity might operate through the development of automaticity. As suggested by dual-process theories (Evans and Stanovich 2013) and by evidence from studies using time-on-task effects, an indicator of automatic processing in a task (Becker et al. 2016; Ćepulić et al. 2020), stimulus type and task difficulty influence the degree of automaticity. In our model (Figure 3), we propose that different cognitive processes, originating from different neural structures, are involved in easier, more automatic tasks, as compared to difficult, more deliberate tasks. 

Putting all these pieces together, we propose that expertise fosters automaticity, thus facilitating resource-efficient local processing rather than the involvement of larger brain networks and higher cognitive functions. Consequently, we suggest investigating the relationship between expertise and face specificity for tasks within a broader difficulty range. If our model holds true in future studies, isolated specificity in easier tasks should be less dependent on the degree of expertise, because automaticity would already be high and the variance limited. In tasks of higher difficulty, however, more expertise might introduce higher automaticity and thus a more pronounced face specificity.

Moreover, task difficulty and the measurement modality of a task are often dependent on each other in that easy task performance is solely measured by RTs, whereas performance in difficult tasks is often solely measured by accuracy rates. This paradigm has its merits, but it leads to a confound between task difficulty and measurement outcome. Consequently, future studies should investigate both accuracy- and speed-based performance along varying task difficulty. Integrated measures of performance that use information on RTs and accuracies simultaneously are required to this purpose, such as the diffusion model (Ratcliff 1978; Wagenmakers et al. 2007). This does not only apply to FC studies. The evidence suggests that easy speed tasks and difficult accuracy tasks are two ends of a continuum of demands in everyday life that serve different purposes from an evolutionary perspective.

### Proposed Differential Functions of Accuracy and Speed in FC

Taking face processing as an ability serving everyday life, the relevance of perceiving and recognizing faces quickly (but perhaps less accurately) or accurately (but perhaps less quickly) represent two ends of a continuum. The evolutionary function of detecting and recognizing faces quickly and automatically may be associated with threat detection: Can I make out a face—whether human or animal—from the surroundings? Is a face familiar and does it belong to a person with good or bad intentions? Does the face belong to an adult, stranger, man, woman or child? All this information is relevant for judging potential threats in the social environment. In contrast, accuracy-based and more deliberate or controlled processes of FC become relevant in everyday situations related with one’s functioning in a social group. Seeing resemblances between faces and being able to memorize and recognize many faces that share the same age and gender help to successfully navigate social relationships. This holds true at all levels of social life, from relationships within families and kin to negotiating conflicting interests and shaping alliances and organizational structures in business, society and politics. From this evolutionary social view on the dichotomy of FC it becomes conceivable that face processing speed vs. accuracy rely on cognitive mechanisms that serve (at least partially) different functions.

## 8. Conclusions

In this review, we addressed two questions: Firstly, is there face specificity also in easy task performance? Secondly, how can face-specific abilities, derived from easy and difficult performance measures, be integrated into the nomological net of socio-cognitive abilities and intelligence? 

To answer the first question, we compared studies on the psychometric structure of speed of FC and concluded that speed in easy tasks is face-specific, but face-specific operations challenged by easy tasks are also *task-specific*, masking specificity in studies where multiple easy speed tasks were jointly modeled. We proposed a cognitive model of face specificity to describe the mechanisms underlying this distinction. Thereby, we further suggest integrating the specific ability to process faces into the nomological net of social cognition, but to heed whether the observed cognitive processes constitute more automatic, easy processing or less automatic, deliberate processing. 

**Figure 4:**
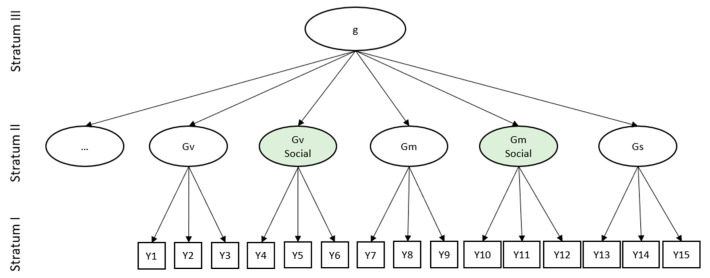
Integration of face memory and perception abilities into the Three-Stratum Model of intelligence (e.g., McGrew 2009). *g*—general factor of cognitive abilities; Gv—general factor of visual processing, i.e., perception; Gv Social—suggested specific factor of perceiving social stimuli in difficult tasks, in our case faces; Gm—general factor of memory; Gm social—suggested specific factor of memorizing faces in difficult tasks; Gs—general mental speed factor. Y1 to Y15—task-derived indicators. Green shades indicate new social factors suggested here.

We suggest that FC is a specific ability that should be subsumed under socio-cognitive abilities. This is supported by the consistent finding of face specificity in accuracy measures across various difficult tasks (e.g., Hildebrandt et al. 2010, 2011, 2013; Wilhelm et al. 2010). Moreover, these studies suggest that FC can be further separated into face memory and face perception. At least the abilities of face memory and face perception constitute separate abilities belonging to FC. FC, in turn, belongs to socio-cognitive abilities. Socio-cognitive abilities and FC have previously been demonstrated to be partly explained by general cognitive abilities, sharing up to 31% of variance with general intelligence (Wilhelm et al. 2010; Wilmer et al. 2010). We suggest integrating these abilities into common taxonomic representations of cognitive abilities, such as the CHC model (McGrew 2009; Figure 4). The CHC model describes Stratum II as a hierarchical layer of specific cognitive abilities. It is Stratum II where we see the integration of face perception and face memory derived from accuracy in difficult tasks as justified (Figure 4). 

Importantly, the structure is different for the speed of FC measured in easy tasks. From studies on the psychometric structure of speed of FC, we can conclude that if multiple speed tasks are jointly modeled together, no face-specific speed factors emerge, as they do for FC accuracy in difficult tasks. Thus, no speed of face memory or speed of face perception exist, nor is there evidence for speed of object perception separately from the speed of FC. Therefore, we suggest integrating the speed of FC not as a separate ability on Stratum II, but to subsume it under the general speed factor already encompassed in three-stratum theory, Gs. However, after reviewing the literature, we conclude that face specificity in easy tasks is not absent. Instead, face specificity in easy tasks becomes unequivocally evident if speed in single tasks is analyzed. This is because the face-specific proportion of variance within one speed task is not related with the face-specific proportion of variance from another task. Therefore, face specificity in speed can be found on Stratum I, on the level of separate tasks. The suggested extension of the three-stratum model with integrated FC abilities is depicted in Figure 4.

We explain the difference in the psychometric structure for easy and difficult tasks by the involvement of different neurocognitive processes. In easy tasks, single face-specific cognitive processes suffice to complete a task (Meyer et al. 2021), probably originating from a small number of face-related neural structures. With increasing difficulty, tasks tap more into task-independent face-specific cognitive abilities (such as face memory or face perception ability). This is consistent with findings of other cognitive tasks that, with increasing difficulty, employ more general cognitive processes such as working memory and more frontal areas of the cortex (Motes et al. 2008; Rypma et al. 2006). 

This distinction likely also plays a role in other abilities from the domain of social cognition besides FC and outside of it. Some potentially specific social cognitive abilities have not yet been extensively studied (e.g., but see Schwartz and Yovel 2016, 2019), for example, the encoding and recall of person-related semantic knowledge or the encoding, retrieval and articulation of person names. Importantly, abilities related to processing expressed emotions (from faces, but also gestures, body language or speech prosody) has not been addressed in the present review. However, these highly relevant social abilities also need further investigation and, potentially, integration as a part of the nomological net of cognitive abilities. Our model (Figure 3) extends to these abilities, predicting that speed in easy tasks and accuracy in difficult tasks might reveal different psychometric structures and should therefore be separated in experimental studies. 

## Figures and Tables

**Table 1:** Examples of easy and difficult face/object memory and perception tasks from Herzmann et al. (2008).

	Task Name	Description	Accuracy (SD)
Easy Memory	Delayed non-matching to sample	Two faces/houses are presented simultaneously. Participants decide which of the two faces/houses does not match a previously memorized sample stimulus.	97% (4%)
Easy Perception	Simultaneous matching of morphed faces/houses	Two faces/houses are presented simultaneously, each morphed from the same two parent faces. Participants decide whether the stimuli are the same or different. In the *same* condition, morphs are derived in equal parts from parent stimuli, in *different* condition at a ratio of 20/80.	93% (6%)
Difficult Memory	Eyewitness testimony	Recognition of distractor faces from a previous task, which had not been required to memorize. In each trial, two faces are presented on the screen, one of which is the target, familiar from a previous task.	65% (11%)
Difficult Perception	Simultaneous matching of spatially manipulated faces	Two faces/houses are presented simultaneously and participants decide whether they are the same or different. In the *different* condition, the spatial relationship between certain features are different; in 50% of the trials, stimuli are presented upside down.	69% (11%)

**Table 2:** Summary of studies investigating face specificity in speed.

Study	Model	Tasks Used in Model	Dependent Variable	Specific Face Factor in Easy Tasks
Hildebrandt et al. (2013)	1	Hard: PW, SM, FR, AC ^1^, DR, EY Easy: RS, DNMS, VP, UH, M	Speed, accuracy	no
Nowparast Rostami et al. (2017)	1	Hard: SM, FR, AC ^1^, DR, EY Easy: DNMS, M, RS, V	Speed	no
Ćepulić et al. (2018)	1	Easy: L/R ^1^ Hard: L/R ^1^	Speed	yes
Meyer et al. (2021)	1	Easy: L/R ^1^	Speed	yes
	2	Easy: DNMS	Speed	yes
	3	Easy: M	Speed	yes
	4	Easy: RS	Speed	yes
	5	Easy: V	Speed	yes

*Note*. Columns *Study* and *Model* identify which model was used from which study: in most cases, only one model was included in the table. From Meyer et al. (2021), 5 models were included and consecutively numbered. Full task names are as follows: Difficult (accuracy) tasks. PW—sequential matching of part-whole faces/houses; SM—simultaneous matching of spatially manipulated faces/houses; FR—facial resemblance; AC—acquisition curve; DR—decay rate of learned faces/houses; EY—eyewitness testimony. RS—recognition speed of learned faces/houses; DNMS—delayed non-matching to sample; VP—simultaneous matching of faces from different viewpoints; UH—simultaneous matching of upper face halves; M—simultaneous matching of morphs; L/R—learning and recognition (similar to acquisition curve). ^1^ AC and L/R denote recognition tasks with a learning and a recognition phase. In the AC task, participants were presented a matrix of 30 stimuli for 2 min before the recognition phase followed. In the L/R tasks’ learning phase, participants were either presented a matrix of 4 stimuli for 12 s (easy condition) or 15 stimuli for 40 s (difficult condition) in Ćepulić et al. (2018) or were presented with a set of 12 (easy condition) or 36 stimuli (difficult condition) sequentially until a learning criterion was reached (Meyer et al. 2021).

## Data Availability

For the present article, studies published elsewhere were reviewed. No new data were collected. Data from some of the studies on which the present review is based can be found at https://osf.io/ndz86/ and https://osf.io/s2t3f/.

## Note

1 A nomological net is a representation of a construct, along with its measurements in which the relationships between the measured variables and their corresponding construct, as well as the relationships between different, more or less similar constructs is being summarized.

2 We refer to mechanisms as the interplay of psychological (or other, for example neural or genetic) processes, reflecting the laws underlying the pattern of interrelations between observed variables. In our use of the term, mechanisms are not necessarily identical with the cause of a phenomenon, but represent a common set of rules underlying the phenomenon.

## References

[B1-jintelligence-09-00030] Adolphs Ralph (2009). The social brain: Neural basis of social knowledge. Annual Review of Psychology.

[B2-jintelligence-09-00030] Bargh John A. (1982). Attention and automaticity in the processing of self-relevant information. Journal of Personality and Social Psychology.

[B3-jintelligence-09-00030] Barton Jason J. S. (2008). Structure and function in acquired prosopagnosia: Lessons from a series of 10 patients with brain damage. Journal of Neuropsychology.

[B4-jintelligence-09-00030] Becker Nicolas, Schmitz Florian, Göritz Anja, Spinath Frank (2016). Sometimes more is better, and sometimes less is better: Task complexity moderates the response time accuracy correlation. Journal of Intelligence.

[B5-jintelligence-09-00030] Bentin Shlomo, Allison Truett, Puce Aina, Perez Erik, McCarthy Gregory (1996). Electrophysiological studies of face perception in humans. Journal of Cognitive Neuroscience.

[B6-jintelligence-09-00030] Bernstein Michal, Yovel Galit (2015). Two neural pathways of face processing: A critical evaluation of current models. Neuroscience and Biobehavioral Reviews.

[B7-jintelligence-09-00030] Bodamer Joachim (1947). Die Prosop-Agnosie. Archiv Fuer Psychiatrie Und Nervenkrankheiten.

[B8-jintelligence-09-00030] Bruce Vicky, Young Andrew (1986). Understanding face recognition. Journal of Clinical Ultrasound.

[B9-jintelligence-09-00030] Bukach Cindy M., Gauthier Isabel, Tarr Michael J. (2006). Beyond faces and modularity: The power of an expertise framework. Trends in Cognitive Sciences.

[B10-jintelligence-09-00030] Burns Edwin J., Arnold Taylor, Bukach Cindy M. (2019). P-curving the fusiform face area: Meta-analyses support the expertise hypothesis. Neuroscience & Biobehavioral Reviews.

[B11-jintelligence-09-00030] Calder Andrew J., Young Andrew W. (2005). Understanding the recognition of facial identity and facial expression. Nature Reviews Neuroscience.

[B12-jintelligence-09-00030] Calder Andrew J., Keane Jill, Young Andrew W., Dean Michael (2000). Configural information in facial expression perception. Journal of Experimental Psychology: Human Perception and Performance.

[B13-jintelligence-09-00030] Carroll John B. (1978). How shall we study individual differences in cognitive abilities?—Methodological and theoretical perspectives. Intelligence.

[B14-jintelligence-09-00030] Carroll John B. (1993). Human Cognitive Abilities: A Survey of Factor—Analytic Studies.

[B15-jintelligence-09-00030] Ćepulić Dominik-Borna, Schmitz Florian, Hildebrandt Andrea (2020). Do time-on-task effects reveal face specificity in object cognition?. Journal of Cognitive Psychology.

[B16-jintelligence-09-00030] Ćepulić Dominik-Borna, Wilhelm Oliver, Sommer Werner, Hildebrandt Andrea (2018). All categories are equal, but some categories are more equal than others: The psychometric structure of object and face cognition. Journal of Experimental Psychology: Learning Memory and Cognition.

[B17-jintelligence-09-00030] Chernorizov Alexander M., Jiang Zhong-qing, Petrakova Anastasia V., Zinchenko Yuri P. (2016). Face cognition in humans: Psychophysiological, developmental, and cross-cultural aspects. Psychology in Russia: State of the Art.

[B18-jintelligence-09-00030] Cheung Olivia S., Gauthier Isabel (2010). Selective Interference on the Holistic Processing of Faces in Working Memory. Journal of Experimental Psychology: Human Perception and Performance.

[B19-jintelligence-09-00030] Cosmides Leda, Tooby John (1994). Beyond intuition and instinct blindness: Toward an evolutionarily rigorous cognitive science. Cognition.

[B20-jintelligence-09-00030] Cross John F., Cross Jane, Daly James (1971). Sex, race, age, and beauty as factors in recognition of faces. Perception & Psychophysics.

[B21-jintelligence-09-00030] Danthiir Vanessa, Roberts Richard D., Schulze Ralf, Wilhelm Oliver (2005). Mental speed: On frameworks, paradigms, and a platform for the future. Handbook of Understanding and Measuring Intelligence.

[B22-jintelligence-09-00030] Diamond Rhea, Carey Susan (1986). Why Faces Are and Are Not Special. An Effect of Expertise. Journal of Experimental Psychology: General.

[B23-jintelligence-09-00030] Donders Frans C. (1969). On the speed of mental processes: Attention and Performance II. Acta Psychologica.

[B24-jintelligence-09-00030] Dowdle Logan T., Ghose Geoffrey, Ugurbil Kamil, Yacoub Essa, Vizioli Luca (2021). Clarifying the role of higher-level cortices in resolving perceptual ambiguity using ultra high field fMRI. NeuroImage.

[B25-jintelligence-09-00030] Doyon Julien, Benali Habib (2005). Reorganization and plasticity in the adult brain during learning of motor skills. Current Opinion in Neurobiology.

[B26-jintelligence-09-00030] Duchaine Brad, Yovel Galit (2015). A Revised Neural Framework for Face Processing. Annual Review of Vision Science.

[B27-jintelligence-09-00030] Duchaine Brad, Nakayama Ken (2006). The Cambridge Face Memory Test: Results for neurologically intact individuals and an investigation of its validity using inverted face stimuli and prosopagnosic participants. Neuropsychologia.

[B28-jintelligence-09-00030] Dunbar Robin I. M. (1998). The social brain hypothesis. Evolutionary Anthropology: Issues, News, and Reviews.

[B29-jintelligence-09-00030] Dunbar Robin I. M. (2003). The social brain: Mind, language, and society in evolutionary perspective. Annual Review of Anthropology.

[B30-jintelligence-09-00030] Dunbar Robin I. M., Shultz Susanne (2007). Evolution in the social brain. Science.

[B31-jintelligence-09-00030] Dunst Beate, Benedek Mathias, Jauk Emanuel, Bergner Sabine, Koschutnig Karl, Sommer Markus, Ischebeck Anja, Spinath Birgit, Arendasy Martin, Bühner Markus (2014). Neural efficiency as a function of task demands. Intelligence.

[B32-jintelligence-09-00030] Eimer Martin (2000). The face-specific N170 component reflects late stages in the structural encoding of faces. NeuroReport.

[B33-jintelligence-09-00030] Evans Jonathan S. B. T., Stanovich Keith E. (2013). Dual-process theories of higher cognition. Perspectives on Psychological Science.

[B34-jintelligence-09-00030] Farah Martha J., Tanaka James W., Drain H. Maxwell (1995). What causes the face inversion effect?. Journal of Experimental Psychology: Human Perception and Performance.

[B35-jintelligence-09-00030] Farah Martha J., Wilson Kevin D., Drain Maxwell, Tanaka James. N. (1998). What is “Special” about face perception?. Psychological Review.

[B36-jintelligence-09-00030] Flanagan Dawn P., McGrew Kevin S. (1998). Interpreting intelligence tests from contemporary Gf-Gc Theory: Joint confirmatory factor Aaalysis of the WJ-R and KAIT in a non-white sample. Journal of School Psychology.

[B37-jintelligence-09-00030] Flanagan Dawn P., Dixon Shauna G., Reynolds Cecil R., Vannest Kimberly J., Fletcher-Janzen Elaine (2014). The Cattell-Horn-Carroll theory of cognitive abilities. Encyclopedia of Special Education.

[B38-jintelligence-09-00030] Garrido Lúcia, Eisner Frank, McGettigan Carolyn, Stewart Lauren, Sauter Disa, Hanley John R., Schweinberger Stefan R., Warren Jason. D., Duchaine Brad (2009). Developmental phonagnosia: A selective deficit of vocal identity recognition. Neuropsychologia.

[B39-jintelligence-09-00030] Gauthier Isabel (2018). Domain-specific and domain-general individual differences in visual object recognition. Current Directions in Psychological Science.

[B40-jintelligence-09-00030] Gauthier Isabel, Bukach Cindy (2007). Should we reject the expertise hypothesis?. Cognition.

[B41-jintelligence-09-00030] Gauthier Isabel, Curby Kim M., Skudlarski Pawel, Epstein Russell A. (2005). Individual differences in FFA activity suggest independent processing at different spatial scales. Cognitive Affective and Behavioral Neuroscience.

[B42-jintelligence-09-00030] Gauthier Isabel, McGugin R. W., Richler J. J., Herzmann Grit, Speegle Magen, Gulick Ana E. Van (2014). Experience moderates overlap between object and face recognition, suggesting a common ability. Journal of Vision.

[B43-jintelligence-09-00030] Goldhammer Frank, Naumann Johannes, Stelter Annette, Tóth Krisztina, Rölke Heiko, Klieme Eckhard (2014). The time on task effect in reading and problem solving is moderated by task difficulty and skill: Insights from a computer-based large-scale assessment. Journal of Educational Psychology.

[B44-jintelligence-09-00030] Gunnery Sarah D., Hall Judith A., Ruben Mollie A. (2013). The deliberate duchenne smile: Individual differences in expressive control. Journal of Nonverbal Behavior.

[B45-jintelligence-09-00030] Guttman Louis, Lazarsfeld Paul F. (1954). A new approach to factor analysis: The Radex. Mathematical Thinking in the Social Sciences.

[B46-jintelligence-09-00030] Hammar Åsa, Seel Norbert M. (2012). Automatic information processing. Encyclopedia of the Sciences of Learning.

[B47-jintelligence-09-00030] Happé Francesca, Cook Jennifer L., Bird Geoffrey (2017). The structure of social cognition: In (ter) dependence of sociocognitive processes. Annual Review of Psychology.

[B48-jintelligence-09-00030] Haxby James V., Hoffman Elizabeth A., Gobbini Ida (2000). The distributed human neural system for face perception. Trends in Cognitive Sciences.

[B49-jintelligence-09-00030] Herzmann Grit, Kunina Olga, Sommer Werner, Wilhelm Oliver (2010). Individual differences in face cognition: Brain-behavior relationships. Journal of Cognitive Neuroscience.

[B50-jintelligence-09-00030] Herzmann Grit, Danthiir Vanessa, Schacht Annekathrin, Sommer Werner, Wilhelm Oliver (2008). Toward a comprehensive test battery for face cognition: Assessment of the tasks. Behavior Research Methods.

[B51-jintelligence-09-00030] Hildebrandt Andrea, Schacht Annekathrin, Sommer Werner, Wilhelm Oliver (2012). Measuring the speed of recognising facially expressed emotions. Cognition & Emotion.

[B52-jintelligence-09-00030] Hildebrandt Andrea, Kiy Astrid, Reuter Martin, Sommer Werner, Wilhelm Oliver (2016). Face and emotion expression processing and the serotonin transporter polymorphism 5-HTTLPR/rs22531. Genes Brain and Behavior.

[B53-jintelligence-09-00030] Hildebrandt Andrea, Wilhelm Oliver, Schmiedek Florian, Herzmann Grit, Sommer Werner (2011). On the specificity of face cognition compared with general cognitive functioning across adult age. Psychology and Aging.

[B54-jintelligence-09-00030] Hildebrandt Andrea, Wilhelm Oliver, Herzmann Grit, Sommer Werner (2013). Face and object cognition across adult age. Psychology and Aging.

[B55-jintelligence-09-00030] Hildebrandt Andrea, Olderbak Sally, Wilhelm Oliver (2015a). Facial emotion expression, Individual differences in. International Encyclopedia of the Social & Behavioral Sciences.

[B56-jintelligence-09-00030] Hildebrandt Andrea, Sommer Werner, Schacht Annekathrin, Wilhelm Oliver (2015b). Perceiving and remembering emotional facial expressions—A basic facet of emotional intelligence. Intelligence.

[B57-jintelligence-09-00030] Hildebrandt Andrea, Sommer Werner, Herzmann Grit, Wilhelm Oliver (2010). Structural Invariance and Age-Related Performance Differences in Face Cognition. Psychology and Aging.

[B58-jintelligence-09-00030] Horn John L., Hofer Scott M., Sternberg Robert J., Berg Cynthia A. (1992). Major abilities and development in the adult period. Intellectual Development.

[B59-jintelligence-09-00030] Kaltwasser Laura, Hildebrandt Andrea, Recio Guillermo, Wilhelm Oliver, Sommer Werner (2014). Neurocognitive mechanisms of individual differences in face cognition: A replication and extension. Cognitive Affective and Behavioral Neuroscience.

[B60-jintelligence-09-00030] Kanwisher Nancy (2000). Domain specificity in face perception. Nature Neuroscience.

[B61-jintelligence-09-00030] Kanwisher Nancy (2010). Functional specificity in the human brain: A window into the functional architecture of the mind. Proceedings of the National Academy of Sciences of the United States of America.

[B62-jintelligence-09-00030] Kanwisher Nancy, Yovel Galit (2006). The fusiform face area: A cortical region specialized for the perception of faces. Philosophical Transactions of the Royal Society B: Biological Sciences.

[B63-jintelligence-09-00030] Kanwisher Nancy, McDermott Josh, Chun Marvin (1997). The fusiform face area: A module in human extrastriate cortex specialized for face perception. Journal of Neuroscience.

[B64-jintelligence-09-00030] Karimi-Rouzbahani Hamid, Ramezani Farzad, Woolgar Alexandra, Rich Aninaq, Ghodrati Masoud (2021). Perceptual difficulty modulates the direction of information flow in familiar face recognition. NeuroImage.

[B65-jintelligence-09-00030] Karmiloff-Smith Annette (1992). Nature, nurture and PDP: Preposterous developmental postulates?. Connection Science.

[B66-jintelligence-09-00030] Karmiloff-Smith Annette, Klima Edward, Bellugi Ursula, Grant Julia, Baron-Cohen Simon (1995). Is there a social module? Language, face processing, and theory of mind in individuals with Williams syndrome. Journal of Cognitive Neuroscience.

[B67-jintelligence-09-00030] Kaufmann Jürgen M., Schweinberger Stefan R., Burton A. Mike (2009). N250 ERP correlates of the acquisition of face representations across different images. Journal of Cognitive Neuroscience.

[B68-jintelligence-09-00030] Keith Timothy Z., Reynolds Matthew R. (2010). Cattell–Horn–Carroll abilities and cognitive tests: What we’ve learned from 20 years of research. Psychology in the Schools.

[B69-jintelligence-09-00030] Kiy Astrid, Wilhelm Oliver, Hildebrandt Andrea, Reuter Martin, Sommer Werner (2013). On the genetic basis of face cognition and its relation to fluid cognitive abilities. Genes Brain and Behavior.

[B70-jintelligence-09-00030] LaBerge David, Samuels S. Jay (1974). Toward a theory of automatic information processing in reading. Cognitive Psychology.

[B71-jintelligence-09-00030] Liu Xinyang, Hildebrandt Andrea, Recio Guillermo, Sommer Werner, Cai Xinxia, Wilhelm Oliver (2017). Individual differences in the speed of facial emotion recognition show little specificity but are strongly related with general mental speed: Psychometric, neural and genetic evidence. Frontiers in Behavioral Neuroscience.

[B72-jintelligence-09-00030] Liu Xinyang, Hildebrandt Andrea, Meyer Kristina, Sommer Werner, Zhou Changsong (2020). Patterns of individual differences in fiber tract integrity of the face processing brain network support neurofunctional models. NeuroImage.

[B73-jintelligence-09-00030] McGrew Kevin S. (2009). CHC theory and the human cognitive abilities project: Standing on the shoulders of the giants of psychometric intelligence research. Intelligence.

[B74-jintelligence-09-00030] McGugin Rankin W., Van Gulick Ana E., Gauthier Isabel (2016). Cortical thickness in fusiform face area predicts face and object recognition performance. Journal of Cognitive Neuroscience.

[B75-jintelligence-09-00030] Meyer Kristina, Garzón Benjamín, Lövdén Martin, Hildebrandt Andrea (2019a). Are global and specific interindividual differences in cortical thickness associated with facets of cognitive abilities, including face cognition?. Royal Society Open Science.

[B76-jintelligence-09-00030] Meyer Kristina, Schmitz Florian, Wilhelm Oliver, Hildebrandt Andrea (2019b). Perceiving faces: Too much, too fast?—Face specificity in response caution. Journal of Experimental Psychology: Human Perception and Performance.

[B77-jintelligence-09-00030] Meyer Kristina, Rostami Hadiseh Nowparast, Ouyang Guang, Debener Stefan, Sommer Werner, Hildebrandt Andrea (2021). Mechanisms of face specificity–differentiating speed and accuracy in face cognition by event-related potentials of central processing. Cortex.

[B78-jintelligence-09-00030] Morton John, Johnson Mark H. (1991). CONSPEC and CONLERN: A two-process theory of infant face recognition. Psychological Review.

[B79-jintelligence-09-00030] Motes Michael A., Malach Rafael, Kozhevnikov Maria (2008). Object-processing neural efficiency differentiates object from spatial visualizers. NeuroReport.

[B80-jintelligence-09-00030] Neta Maital, Whalen Paul J. (2011). Individual differences in neural activity during a facial expression vs. identity working memory task. NeuroImage.

[B81-jintelligence-09-00030] Neubauer Aljoscha C., Fink Andreas (2009). Intelligence and neural efficiency. Neuroscience and Biobehavioral Reviews.

[B82-jintelligence-09-00030] Neumann Markus F., Schweinberger Stefan R. (2008). N250r and N400 ERP correlates of immediate famous face repetition are independent of perceptual load. Brain Research.

[B83-jintelligence-09-00030] Neuner Frank, Schweinberger Stefan R. (2000). Neuropsychological impairments in the recognition of faces, voices, and personal names. Brain and Cognition.

[B84-jintelligence-09-00030] Nowparast Rostami Hadiseh, Sommer Werner, Zhou C., Wilhelm Oliver, Hildebrandt Andrea (2017). Structural encoding processes contribute to individual differences in face and object cognition: Inferences from psychometric test performance and event-related brain potentials. Cortex.

[B85-jintelligence-09-00030] O’Sullivan Maureen, Guilford Joy P. (1975). Six factors of behavioral cognition: Understanding other people. Journal of Educational Measurement.

[B86-jintelligence-09-00030] Oberauer Klaus, Süß Heinz-Martin, Wilhelm Oliver, Wittman Werner W. (2003). The multiple faces of working memory: Storage, processing, supervision, and coordination. Intelligence.

[B87-jintelligence-09-00030] Oberauer Klaus, Süß Heinz-Martin, Schulze Ralf, Wilhelm Oliver, Wittmann Werner W. (2000). Working memory capacity—Facets of a cognitive ability construct. Personality and Individual Differences.

[B88-jintelligence-09-00030] Oberauer Klaus, Wilhelm Oliver, Schmiedek Florian, Beauducel André, Biehl Bernhard, Bosnjak Michael, Conrad Wolgang, Schönberger Gisela, Wagener Dietrich (2005). Experimental strategies in multivariate research. Multivariate Research Strategies: Festschrift in Honor of Werner W. Wittmann.

[B89-jintelligence-09-00030] Olderbak Sally, Hildebrandt Andrea, Wilhelm Oliver (2015). Examining age-related shared variance between face cognition, vision, and self-reported physical health: A test of the common cause hypothesis for social cognition. Frontiers in Psychology.

[B90-jintelligence-09-00030] Olderbak Sally, Wilhelm Oliver, Hildebrandt Andrea, Quoidbach Jordi (2019). Sex differences in facial emotion perception ability across the lifespan. Cognition and Emotion.

[B91-jintelligence-09-00030] Ouyang Guang, Hildebrandt Andrea, Schmitz Florian, Herrmann Christoph S. (2020). Decomposing alpha and 1/f brain activities reveals their differential associations with cognitive processing speed. NeuroImage.

[B92-jintelligence-09-00030] Quinones Sanchez Juan F., Liu Xinyang, Zhou Changsong, Hildebrandt Andrea (2021). Nature and Nurture Shape Structural Connectivity in the Face Processing Brain Network. NeuroImage.

[B93-jintelligence-09-00030] Ratcliff Roger (1978). A theory of memory retrieval. Psychological Review.

[B94-jintelligence-09-00030] Reingold Eyal M., Charness Neil, Schultetus Richard S., Stampe Dave M. (2001). Perceptual automaticity in expert chess players: Parallel encoding of chess relations. Psychonomic Bulletin & Review.

[B95-jintelligence-09-00030] Richler Jennifer J., Gauthier Isabel (2014). A Meta-Analysis and Review of Holistic Face Processing. Psychological Bulletin.

[B96-jintelligence-09-00030] Roberts Richard D., Stankov Lazar (1999). Individual differences in speed of mental processing and human cognitive abilities: Toward a taxonomic model. Learning and Individual Differences.

[B97-jintelligence-09-00030] Rossion Bruno, Caldara Roberto, Seghier Mohamed, Schuller Anne-Marie, Lazeyras Francois, Mayer Eugene (2003). A network of occipito-temporal face-sensitive areas besides the right middle fusiform gyrus is necessary for normal face processing. Brain.

[B98-jintelligence-09-00030] Rotshtein Pia, Geng Joy J., Driver Jon, Dolan Raymond J. (2007). Role of features and second-order spatial relations in face discrimination, face recognition, and individual face skills: Behavioral and functional magnetic resonance imaging data. Journal of Cognitive Neuroscience.

[B99-jintelligence-09-00030] Rypma Bart, Berger Jeffrey S., Prabhakaran Vivek, Bly Benjamin Martin, Kimberg Daniel Y., Biswal Bharat B., D’Esposito Mark (2006). Neural correlates of cognitive efficiency. NeuroImage.

[B100-jintelligence-09-00030] Said Christopher P., Moore Christopher D., Engell Andrew D., Todorov Alexander, Haxby James V. (2010). Distributed representations of dynamic facial expressions in the superior temporal sulcus. Journal of Vision.

[B101-jintelligence-09-00030] Schneider W. Joel, McGrew Kevin S., Flanagan Dawn P., Harrison Patti L. (2012). The Cattell-Horn-Carroll model of intelligence. Contemporary Intellectual Assessment: Theories Tests and Issues.

[B102-jintelligence-09-00030] Schulze Ralf, Wilhelm Oliver, Engle Randall W. (2005). Modeling structures of intelligence. Handbook of Understanding and Measuring Intelligence.

[B103-jintelligence-09-00030] Schwartz Linoy, Yovel Galit (2016). The roles of perceptual and conceptual information in face recognition. Journal of Experimental Psychology: General.

[B104-jintelligence-09-00030] Schwartz Linoy, Yovel Galit (2019). Learning faces as concepts rather than percepts improves face recognition. Journal of Experimental Psychology: Learning Memory and Cognition.

[B105-jintelligence-09-00030] Schweinberger Stefan R., Burton A. Mike (2011). Person perception 25 years after Bruce and Young (1986): An introduction. British Journal of Psychology.

[B106-jintelligence-09-00030] Schweinberger Stefan R., Schneider Dana (2014). Person perception and social cognition. Psychologische Rundschau.

[B107-jintelligence-09-00030] Shakeshaft Nicholas G., Plomin Robert (2015). Genetic specificity of face recognition. Proceedings of the National Academy of Sciences.

[B108-jintelligence-09-00030] Shallice Tim (1988). From Neuropsychology to Mental Structure.

[B109-jintelligence-09-00030] Skuse David H., Lori Adriana, Cubells Joseph F., Lee Irene, Conneely Karen N., Puura Kaija, Lehtimäki Terho, Binder Elisabeth B., Young Larry J. (2014). Common polymorphism in the oxytocin receptor gene (OXTR) is associated with human social recognition skills. Proceedings of the National Academy of Sciences of the United States of America.

[B110-jintelligence-09-00030] Sommer Werner, Hildebrandt Andrea, Kunina-Habenicht Ologa, Schacht Annekathrin, Wilhelm Oliver (2013). Sex differences in face cognition. Acta Psychologica.

[B111-jintelligence-09-00030] Sperling George, Dosher Barbara A., Boff Kenneth R., Kaufman Lloyd, Thomas James P. (1986). Strategy and optimization in human information processing. Handbook of Perception and Human Performance.

[B112-jintelligence-09-00030] Stanovich Keith E., West Richard F., Toplak Maggie E. (2011). The complexity of developmental predictions from dual process models. Developmental Review.

[B113-jintelligence-09-00030] Sternberg Saul (1969). The discovery of processing stages: Extensions of Donders’ method. Acta Psychologica.

[B114-jintelligence-09-00030] Süß Heinz-Martin, Beauducel Andre, Wilhelm Oliver, Engle Randall W. (2005). Faceted models of intelligence. Understanding and Measuring Intelligence.

[B115-jintelligence-09-00030] Tanaka James W., Simonyi Diana (2016). The “Parts and Wholes” of face recognition: A review of the literature. Quarterly Journal of Experimental Psychology.

[B116-jintelligence-09-00030] Tanaka James W., Gordon Iris, Rhodes Gillian, Calder Andrew, Johnson Mark, Haxby James V. (2011). Features, configuration, and holistic face processing. The Oxford Handbook of Face Perception.

[B117-jintelligence-09-00030] Tanaka James W., Gauthier Isabel (1997). Expertise in object and face recognition. Psychology of Learning and Motivation.

[B118-jintelligence-09-00030] Tanaka James W., Farah Martha J. (1993). Parts and wholes in face recognition. The Quarterly Journal of Experimental Psychology Section A.

[B119-jintelligence-09-00030] Thorndike Edward L. (1920). Intelligence and its uses. Harper’s Magazine.

[B120-jintelligence-09-00030] Turano Maria T., Viggiano Maria P. (2017). The relationship between face recognition ability and socioemotional functioning throughout adulthood. Aging Neuropsychology and Cognition.

[B121-jintelligence-09-00030] Valentine Tim (1988). Upside-down faces: A review of the effect of inversion upon face recognition. British Journal of Psychology.

[B122-jintelligence-09-00030] Verhallen Roeland J., Bosten Jenny M., Goodbourn Patrick T., Lawrance-Owen Adam J., Bargary Gary, Mollon John D. (2017). The Oxytocin receptor gene (OXTR) and face recognition. Psychological Science.

[B123-jintelligence-09-00030] Vernon Philip E. (1933). Some characteristics of the good judge of personality. Journal of Social Psychology.

[B124-jintelligence-09-00030] Voss Andreas, Rothermund Klaus, Voss Jochen (2004). Interpreting the parameters of the diffusion model: An empirical validation. Memory & Cognition.

[B125-jintelligence-09-00030] Wagenmakers Eric-Jan, Maas Han L. J. Van Der, Grasman Raoul P. P. P. (2007). An EZ-diffusion model for response time and accuracy. Psychonomic Bulletin and Review.

[B126-jintelligence-09-00030] Wang Yin, Metoki Athanasia, Smith David V., Medaglia John D., Zang Yinyin, Benear Susan, Popal Haroon, Lin Ying, Olson Ingrid R. (2020). Multimodal mapping of the face connectome. Nature Human Behaviour.

[B127-jintelligence-09-00030] Wechsler D. (1958). The Measurement and Appraisal of Adult Intelligence.

[B128-jintelligence-09-00030] Wilhelm Oliver, Hildebrandt Andrea, Manske Karsten, Schacht Annekathrin, Sommer Werner (2014). Test battery for measuring the perception and recognition of facial expressions of emotion. Frontiers in Psychology.

[B129-jintelligence-09-00030] Wilhelm Oliver, Herzmann Grit, Kunina Olga, Sommer Werner (2007). Face cognition: A set of distinct mental Abilities. Nature Precedings.

[B130-jintelligence-09-00030] Wilhelm Oliver, Herzmann Grit, Kunina Olga, Danthiir Vanessa, Schacht Annekathrin, Sommer Werner (2010). Individual differences in perceiving and recognizing faces-One element of social cognition. Journal of Personality and Social Psychology.

[B131-jintelligence-09-00030] Wilmer Jeremy B. (2017). Individual differences in face recognition: A decade of discovery. Current Directions in Psychological Science.

[B132-jintelligence-09-00030] Wilmer Jeremy B., Germine Laura, Chabris Christopher F., Chatterjee Garga, Gerbasi Margaret, Nakayama Ken (2012). Capturing specific abilities as a window into human individuality: The example of face recognition. Cognitive Neuropsychology.

[B133-jintelligence-09-00030] Wilmer Jeremy B., Germine Laura, Chabris Christopher F., Chatterjee Garga, Williams Mark, Loken Eric, Nakayama Ken, Duchaine Bradley (2010). Human face recognition ability is specific and highly heritable. Proceedings of the National Academy of Sciences of the United States of America.

[B134-jintelligence-09-00030] Wilmer Jeremy B., Germine Laura T., Nakayama Ken (2014). Face recognition: A model specific ability. Frontiers in Human Neuroscience.

[B135-jintelligence-09-00030] Young Andrew W., Hellawell Deborah, Hay Dennis C. (1987). Configurational information in face perception. Perception.

[B136-jintelligence-09-00030] Young Andrew W., Newcombe Freda, de Haan Edward H. F., Small Marian, Hay Dennis C. (1993). Face perception after brain injury. Brain.

[B137-jintelligence-09-00030] Yovel Galit, Wilmer Jeremy B., Duchaine Bradley (2014). What can individual differences reveal about face processing?. Frontiers in Human Neuroscience.

[B138-jintelligence-09-00030] Zhang Yang, Wang Yue (2007). Neural plasticity in speech acquisition and learning. Bilingualism.

[B139-jintelligence-09-00030] Zhu Qi, Song Yiying, Hu Siyua, Li Xiaobai, Tian Moqian, Zhen Zonglei, Dong Qi, Kanwisher Nany, Liu Jia (2010). Heritability of the specific cognitive ability of face perception. Current Biology.

